# Hyaluronidase Inhibitory Activity of Pentacylic Triterpenoids from *Prismatomeris tetrandra* (Roxb.) K. Schum: Isolation, Synthesis and QSAR Study

**DOI:** 10.3390/ijms17020143

**Published:** 2016-02-14

**Authors:** Nor Hayati Abdullah, Noel Francis Thomas, Yasodha Sivasothy, Vannajan Sanghiran Lee, Sook Yee Liew, Ibrahim Ali Noorbatcha, Khalijah Awang

**Affiliations:** 1 Natural Product Division, Forest Research Institute Malaysia, 52109 Kepong, Malaysia; norhayatiab@frim.gov.my; 2 Department of Chemistry, Faculty of Science, University of Malaya, 50603 Kuala Lumpur, Malaysia; noelfthomas@um.edu.my (N.F.T.); yasodha@um.edu.my (Y.S.); vannajan@um.edu.my (V.S.L.); joeyliew5382@um.edu.my (S.Y.L.); 3 Centre for Natural Products and Drug Discovery (CENAR), University of Malaya, 50603 Kuala Lumpur, Malaysia; 4 BioProcess and Molecular Engineering Research Unit (BPMERU), Department of Biotechnology Engineering, Faculty of Engineering, International Islamic University Malaysia, 53100 Kuala Lumpur, Malaysia; ibrahiman@iium.edu.my

**Keywords:** hyaluronidase, ursolic acid, QSAR, semi empirical quantum chemical, molecular docking, *Prismatomeris tetrandra* (Roxb.) K. Schum, Rubiaceae

## Abstract

The mammalian hyaluronidase degrades hyaluronic acid by the cleavage of the β-1,4-glycosidic bond furnishing a tetrasaccharide molecule as the main product which is a highly angiogenic and potent inducer of inflammatory cytokines. Ursolic acid **1**, isolated from *Prismatomeris tetrandra*, was identified as having the potential to develop inhibitors of hyaluronidase. A series of ursolic acid analogues were either synthesized via structure modification of ursolic acid **1** or commercially obtained. The evaluation of the inhibitory activity of these compounds on the hyaluronidase enzyme was conducted. Several structural, topological and quantum chemical descriptors for these compounds were calculated using semi empirical quantum chemical methods. A quantitative structure activity relationship study (QSAR) was performed to correlate these descriptors with the hyaluronidase inhibitory activity. The statistical characteristics provided by the best multi linear model (BML) (*R*^2^ = 0.9717, *R*^2^_cv_ = 0.9506) indicated satisfactory stability and predictive ability of the developed model. The *in silico* molecular docking study which was used to determine the binding interactions revealed that the ursolic acid analog **22** had a strong affinity towards human hyaluronidase.

## 1. Introduction

Hyaluronic acid (HA) is a polymer of varying chain length composed of a repeating dissacharide unit, *N*-acetylhyaluronic acid, linked via the hexosaminidic bonds in β-(1→4) linkages [1]. It usually consists of 2000–2500 dissacharides to give a molecular mass between 10^6^ to 10^7^ Da and extended lengths of 2–25 µm [2]. It can be found in an extracellular matrix, especially in soft connective tissues of all vertebrates and in the capsule of some bacteria [2]. HA plays an important role in biological processes such as cellular adhesion, mobility differentiation processes that act as lubricant and shock absorber, regulates water balance and osmotic pressure [3]. It is also the structure-forming molecule in the vitreous humor of the eye, in Wharton’s jelly and in joint fluids.

Hyaluronidase degrades HA by cleaving the *N*-acetylglucosamidic bonds of HA via a β-elimination process to produce HA oligosaccharides, with chain lengths of four to 25 disaccharides which possess angiogenic properties [3,4]. The process of HA oligosaccharide formation favors the production of new blood vessels, thus facilitating the development of cancer tumors. Since this enzyme has been implicated in many biological functions, such as allergy, inflammation, migration of cancer cells and permeability of the vascular system, the modulation of hyaluronidases by suitable inhibitors will be useful for normal homeostasis in the body.

*Prismatomeris tetrandra* (Roxb.) K. Schum is also a synonym for *P. malayana* Ridley and *P. albidiflora* King [5]. *P. tetrandra* is popular in Malaysia as “tongkat haji samat”. This plant is distributed in South East Asia. It is traditionally used to treat wounds, bronchitis and snakebites [6]. In this study, three pentacylic triterpenoids (PT) with hyaluronidase inhibitory activity namely, ursolic acid **1**, 3β,19,23-trihydroxyurs-12-en-28-oic acid **2** and 3β-acetylolean-12-en-28-oic acid **3** were isolated.

PTs are aglycones of saponins and exist abundantly in the plant kingdom. They have a wide range of activities such as cytotoxicity and anti-microbial, anti-oxidant, anti-HIV properties [7,8,9,10]. Ursolic acid **1** and several other PTs have been also reported to possess a wide range of anti-inflammatory activities. Their systemic anti-inflammatory effects might be due to their actions on the mediators signaling such as on histamine, human leukocyte elastase, cytokines, reactive oxygen species, lipid peroxidation and lipid-derived mediators [11]. Besides that, some PTs have also been reported to show hepatoprotective activity, inhibit edema in animal models and immune modulating actions in mice.

Structural modification studies on PTs have been reported for betulinic acid and ursolic acid in order to investigate their potential as anti-tumor drugs [12,13,14,15,16]. The potential of PTs and their derivatives on anti-HIV inhibition towards HIV protease and cytotoxicity on tumor cell lines have also been studied [10,17,18,19,20]. However, when compared to the other bioactivity studies, ursolic acid **1** and its derivatives have never been thoroughly explored for their anti-inflammatory properties, specifically on the inhibition activity towards hyaluronidase.

Several quantitative structure activity relationship (QSAR) studies have been conducted on PT compounds based on inhibition towards glycogen phosphorylase, and anti-cancer, immunomodulatory, and anti HIV activities [21,22,23,24]. However, the QSAR study on PTs including ursolic acid and its derivatives as anti-inflammatory agents, due to hyaluronidase inhibitory activity, has not been reported.

In this work, we report the isolation and characterization of natural PTs including ursolic acid, and also the synthesis of seven analogues of ursolic acid. In addition, all PTs together with twenty ursolic acid analogues were subjected to hyaluronidase inhibitory assay. The results were then used to build QSAR models based on the quantum chemical descriptors which were calculated from the three dimensional structure of the PTs. The computer software CODESSA 2.6 was used in this study to build the QSAR model. In order to investigate the influence of different descriptors on the hyaluronidase inhibitory ability of PTs, both the Heuristic and Best Multi Linear model (BML) were used to develop a multivariable linear model. Thus, the objective of this study was to understand the inhibition towards hyaluronidase activity by the PTs with a wide range of structures. Molecular docking was performed to predict the complex structure and determine the binding mode of interaction with hyaluronidase. The new and accurate QSAR model established in this study can be used to predict the activity. A predicted compound (PTC A) using the QSAR model developed was also proposed.

## 2. Results and Discussion

### 2.1. Isolation and Characterization of Triterpenoids **1**–**3**

A total of three PTs were isolated from *P. tetrandra*. The two PTs which were obtained from the chloroform (CHCl_3_) fraction were identified as 3β-urs-12-en-28-oic acid **1** and 3β,19,23-trihydroxyurs-12-en-28-oic acid **2**. The PTs isolated from the roots were characterized as 3β-acetylolean-12-en-28-oic acid **3**.

### 2.2. Synthesis

**Figure 1:**
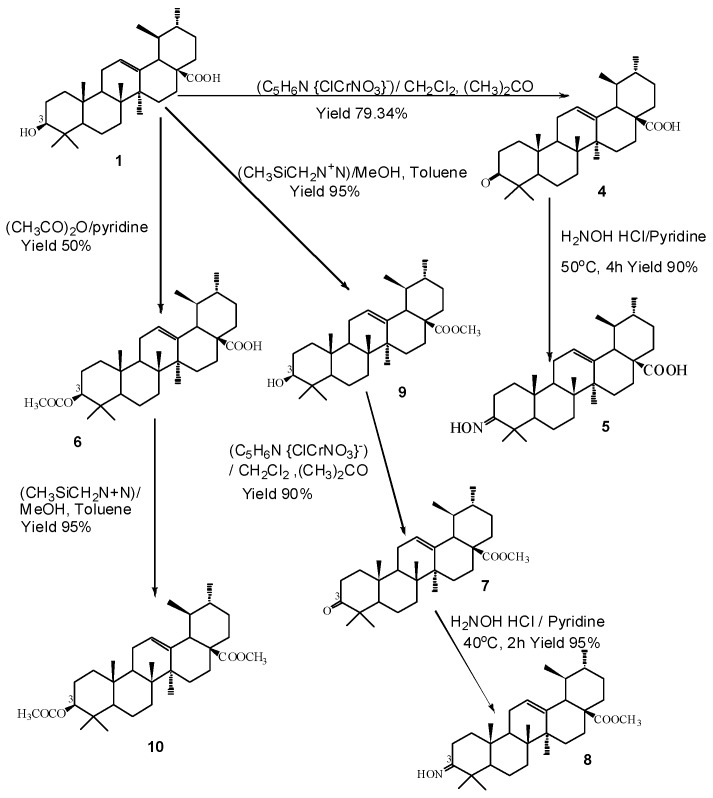
Synthesis of ursolic acid **1** derivatives with different substituents at C-3. ((C_5_H_6_N {ClCrNO_3_}) = Pyridinium chlorochromate; CH_2_Cl_2_ = dichloromethane; (CH_3_)_2_CO = acetone; H_2_NOHHCl = hydroxylamine hydrochloride; (CH_3_CO)_2_ = acetic anhydride; (CH_3_SiCH_2_N^+^N) = trimethylsilyl diazomethane (TMS)).

Compounds **4**–**10** were synthesized from ursolic acid **1**. Structural modifications were carried out at positions C-3 and C-28 of ursolic acid **1** (Figure 1). The modifications were carried out either by acetylation, methylation or amino group introduction or by combination of the three methods. Compounds **11**–**30** were purchased from Chromadex^®^ which was obtained from natural sources *i.e.*, plants. The structures were confirmed on the basis of their ^1^H NMR, ^13^C NMR, and ESI-MS spectroscopy data and upon comparison with literature values [25,26].

### 2.3. Hyaluronidase Inhibitory Activity

The study was conducted on compounds **1**–**30**. The assay was performed according to the modified Sigma protocol [27]. The IC_50_ values for the inhibitory activities of compounds **1**–**30** are presented in Table 1. The results showed that out of the 30 compounds, only 24 were found to exhibit activity below 2000 µM. Oleanolic acid methyl ester **16** (84.52 µM) and carbonexolone **22** (56.33 µM) showed higher activities compared to ursolic acid **1**. Compounds **4** (162.83 µM), **5** (190.94 µM), **6** (136.92 µM), **9** (182.51 µM), **13** (206.21 µM), **14** (211.44 µM), **15** (140.91 µM), **19** (115.96 µM) and **21** (146.18 µM) showed higher activities compared to apigenin, the positive control. Compounds **25**–**30** on the other hand were inactive as their inhibitory activities were less than 20% at concentrations of up to 2 × 10^3^ µM (Table 1). All compounds were prepared at an initial concentration of 2000 µM before serial dilution. Thus, if the inhibition towards hyaluronidase at 2000 µM was less than 20%, the compound will be considered as inactive. The highest concentration was prepared less than 2000 µM to avoid the solubility problem.

**Table 1:** Hyaluronidase inhibitory activity of ursolic acid **1** and its analogues at the concentration of 100–2000 µM.

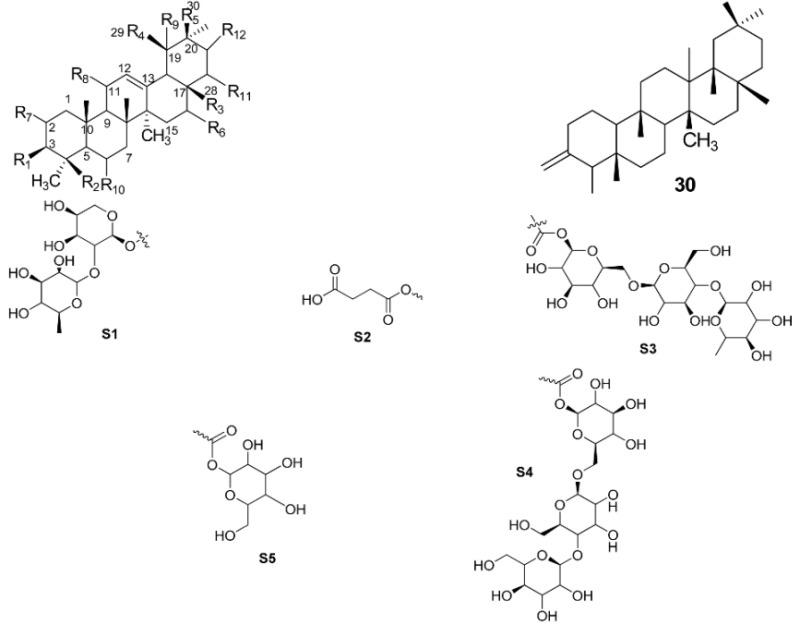													
Substitutional Pattern													^a^ IC_50_
Compounds	R1	R2	R3	R4	R5	R6	R7	R8	R9	R10	R11	R12	
1	OH	CH_3_	COOH	CH_3_	H	H	H	H	H	H	H	H	103.18 ± 1.70 **
2	OH	CH_2_OH	COOH	CH_3_	H	H	H	H	OH	H	H	H	286.95 ± 10.28
3	OAc	CH_3_	COOH	H	CH_3_	H	H	H	H	H	H	H	1466.5 ± 2.37
4	=O	CH_3_	COOH	CH_3_	H	H	H	H	H	H	H	H	162.83 ± 6.37 *
5	NOH	CH_3_	COOH	CH_3_	H	H	H	H	H	H	H	H	190.94 ± 0.01 *
6	OAc	CH_3_	COOH	CH_3_	H	H	H	H	H	H	H	H	136.92 ± 0.04 *
7	=O	CH_3_	COOCH_3_	CH_3_	H	H	H	H	H	H	H	H	1184.15 ± 6.63
8	NOH	CH_3_	COOCH_3_	CH_3_	H	H	H	H	H	H	H	H	275.68 ± 1.42
9	OH	CH_3_	COOCH_3_	CH_3_	H	H	H	H	H	H	H	H	182.51 ± 0.84 *
10	OAc	CH_3_	COOCH_3_	CH_3_	H	H	H	H	H	H	H	H	812.93 ± 10.29
11	OH	CH_3_	CH_3_	H	COOCH_3_	H	H	=O	H	H	H	H	1750.91 ± 2.38
12	OH	CH_2_OH	CH_3_	CH_3_	H	H	H	H	H	H	H	H	227.97 ± 2.81
13	OH	CH_3_	CH_2_OH	H	CH_3_	H	H	H	H	H	H	H	206.21 ± 2.32
14	OH	CH_3_	CH_3_	CH_3_	H	H	H	H	H	H	H	H	211.44 ± 3.16 *
15	OH	CH_3_	COOH	H	CH_3_	OH	H	H	H	H	H	H	140.91 ± 6.71
16	OH	CH_3_	COOCH_3_	H	CH_3_	H	H	H	H	H	H	H	84.52 ± 0.01 **
17	S1	CH_2_OH	COOH	H	CH_3_	H	H	H	H	H	H	H	842.54 ± 0.11
18	OH	CH_3_	CH_3_	H	CH_3_	H	H	H	H	H	H	H	215.66 ± 4.27 *
19	OH	CH_2_OH	COOH	CH_3_	H	H	OH	H	H	H	H	H	115.96 ± 0.47 *
20	OH	CH_3_	COOH	H	CH_3_	H	H	H	H	H	H	H	227.97 ± 5.99
21	OH	CH_3_	CH_3_	H	COOH	H	H	=O	H	H	H	H	146.18 ± 2.67 *
22	S2	CH_3_	CH_3_	H	COOH	H	H	=O	H	H	H	H	56.33 ± 0.01 **
23	OH	COOH	CH_3_	H	CH_3_	H	H	H	H	H	H	H	1482.56 ± 0.70
24	OH	CH_2_OH	COOH	H	CH_3_	H	H	H	H	H	H	H	230.00 ± 2.17
25	*O*-glucoside	CH_3_	COOH	H	CH_3_	OH	H	H	H	H	H	H	NA
26	OH	COOH	CH_3_	CH_3_	H	H	H	H	H	H	H	H	NA
27	OH	S3	CH_2_OH	CH_3_	H	H	OH	H	H	H	H	H	NA
28	OH	CH_2_OH	CH_2_OH	H	CH_3_	OH	H	H	H	H	OH	OH	NA
29	OH	CH_2_OH	COOS4	CH_3_	H	H	OH	H	H	OH	H	H	NA
30	–	–	–	–	–	–	–	–	–	–	–	–	NA
Apigenin	–	–	–	–	–	–	–	–	–	–	–	–	214.74

^a^ NA-inhibitory activity, 20% at concentration up to 2000 µM; positive control-Apigenin. Values were presented as the mean of three independent experiments performed in triplicate; * Mean for percentage inhibition were significantly different (one-way analysis of variance, *p* < 0.05); ** Mean for percentage inhibition were significantly different (one-way analysis of variance, *p* < 0.005).

### 2.4. Structure Activity Relationship (SAR) of Ursolic Acid **1** and Its Analogues

Basically, the analogues are classified into two pentacyclic triterpene (PTC) skeletons; ursane (**1**, **2**, **4**, **5**, **6**, **7**, **8**, **9**, **10**, **12**, **14**, **19**, **26**, **27**, **29**) and oleanane (**3**, **13**, **15**, **16**, **17**, **18**, **20**, **23**, **24**, **25**, **28**, **30**). The results in Table 1 showed that ursolic acid **1** was more active than oleanolic acid **20**. However, the comparison between the analogues or derivatives with the similar skeletons such as **12** and **13**, or **14** and **18**, does not reveal a large difference in their activity. Thus, it showed that the geminal or vicinal arrangement of the methyl-29 and 30 did not give a large effect on the activity but with some exception. The discussion will be divided into the ursane and oleanane skeletons.

For the oleanane skeleton, the activity reduced slightly when the methylhydroxyl group was introduced at C-23 (**21** *vs.* **25**). However, the activity increased when the methyl group was introduced at C-17 (**18** *vs.* **20**). The activity was also increased when the hydroxyl group was introduced at C-16 (**15** *vs.* **20**). The C-30 ester derivatives resulted in a great loss of activity (**11** *vs.* **21**), while the carboxylation of the same carbon (C-30) increased the activity (**21**).

Acetylation of 3-OH decreased the inhibitory ability (**3**). Introduction of a sugar moiety with a glycosidic bond to 3-OH or 28-COOH would either reduce the activity drastically (**17**) or become inactive, whereas the addition of an oxo group to C-11 either did not improve the activity or reduced it slightly (**21** *vs.* **22** *vs.* **11**). Too many hydroxyl groups as in **28** also resulted in a loss of activity. Significant improvement in the activity was observed when a methylester group was introduced at C-17 (**16** *vs.* **20**, **18**) or when a carboxypropanoyloxy group, S2, was introduced at C-3 (**22**).

The inhibitory activity of the ursane skeleton analogues decreased for the 3-oxo, 3-hydroxyimino and 3-acetylate derivatives (**4**, **5**, **6**) compared to ursolic acid **1**. A similar trend was observed when the 28-OH was substituted with a methyl group (**5** *vs.* **8**, **1** *vs.* **9**, **6** *vs.* **10**). This observation suggested that the hydroxyl groups at C-3 and C-28 were essential for the hyaluronidase inhibitory activity. The substitution of a methyl group with a carboxyl group at C-23 decreased the inhibitory activity (**14** *vs.* **26**, NA). The introduction of a hydroxymethylene group at C-23 and the addition of a hydroxyl group at C-1 did not affect the activity very much (**19** *vs.* **26**, NA), however, the addition of a hydroxyl group at C-19 further decreased the activity (**2**). The position of the hydroxyl group also affected the activity as it decreased the activity for **2** (C-19) compared to **19** (C-2). The sugar moiety, as usual, would result in a loss of activity (**27**, **29**).

**Figure 2:**
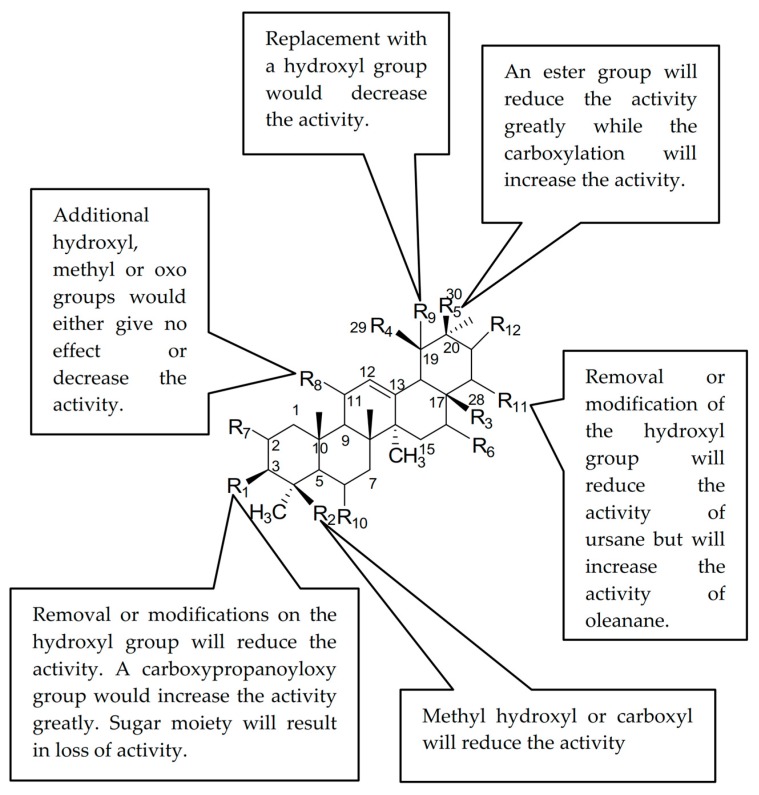
Structure activity relationship of pentacyclic triterpenes (PTs).

It can be concluded that the presence of 3-OH is important for both ursane and oleanane skeletons to inhibit hyaluronidase. Introducing a methyl ester group at C-17 to replace the carboxylic functional group resulted in a different effect on the hyaluronidase inhibition ability of both types of skeletons. Replacement of the 3-OH with hydroxyimino, acetyl and methyl groups lowered the activity. Addition of hydroxyl groups at C-2, C-1 and C-19 or oxo groups at C-11 and C-3, however, would either give no effect or decrease the activity. Introducing a sugar moiety at any position would result in a loss of activity. This could probably be due to the bulkiness of the structure that led to the compounds being unable to reach the active site of the target, which was in the hyaluronidase enzyme. However, the carboxypropionylox group, S2, that replaced the 3-OH, increased the activity. Figure 2 summarizes the structure activity relationship of the PTC compounds (**1**–**30**).

Even though these simplistic structure activity relations are useful for a single functional group substitutional level, it is not easy to predict the cumulative effect of several substitutions on the hyaluronidase inhibition activity using these relations. Hence, it is necessary to adopt the QSAR approach to analyze and predict the effect of substitution on the activity.

### 2.5. QSAR Model and Its Interpretation

The data set from the hyaluronidase inhibitory activity for compounds **1**–**24** were converted into log IC_50_ in order to improve the normal distribution of the experimental data points [28]. After analyzing the data set using the Heuristic method and Best Multi Linear regression method (BML), it was found that the QSAR model from the BML method was statistically robust compared to the Heuristic method.

The data was divided into the test set (8, 11, 19, 6) and the remaining data into the training set. From the data set which consisted of 20 compounds, eleven models were obtained which consisted of two to twelve descriptors as listed in Table 2. The *R*^2^ values as well as the other statistical values also improved (close to 1). This method managed to avoid over fitting of the regression equations by monitoring the increase of *R*^2^ in the equations with successive number of descriptors involved. The procedure is called the break point technique [28]. The procedure was stopped when the difference between *R*^2^ of the two consequent regression equations was less than or equal to 0.02 [28]. However, the best optimum correlation should have the ratio of the data set compounds to the descriptor at 5:1 [29]. Meaning that, there is a descriptor that represents five data points. Thus, from the data set, the optimum descriptor number was four.

**Figure 3:**
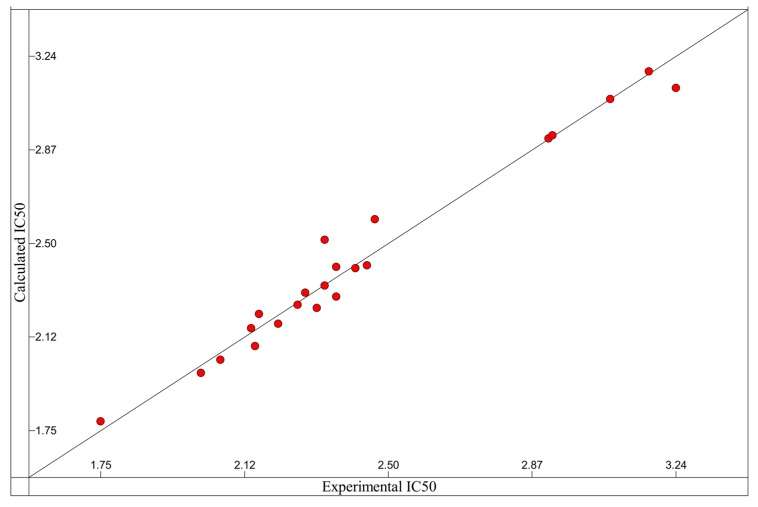
Comparison of the experimental hyaluronidase activity with the activity presented by the QSAR Equation (1), *n* = 20, with *R*^2^ = 0.8579; *s*^2^ = 0.0246; *F* = 21.13; four descriptors.

The best QSAR model was developed using four descriptors. The *p* value is less than 0.01 for each descriptor involved in the model generation. These descriptors were selected, as the addition of more descriptors does not lead to any significant improvement in the correlation. A plot of the experimental *vs.* predicted IC_50_ values is depicted in Figure 3 for the 20 PTs (**1**–**5**, **7**, **9**, **10**, **13**, **24**).

**Table 2:** The best two to twelve descriptors correlation using the (Best Multi Linear) BML method for training set data.

Descriptor Number	Correlation Coefficient (*R*^2^)	Fisher Criteria (*F*)	Standard Deviation (*s*^2^)
2	0.6774	21.13	0.0633
3	0.7992	19.90	0.0373
4	0.8579	16.80	0.026
5	0.8821	19.45	0.0167
6	0.9303	26.71	0.0123
7	0.9591	36.85	0.0065
8	0.9866	92.38	0.0037
9	0.9934	150.00	0.0021
10	0.9966	237.73	0.0010
11	0.9994	1020.35	0.0005
12	0.9998	3234.68	0.0002

The QSAR equation relating the seven descriptors to the hyaluronidase inhibitory activity is given below:

Log(1/IC_50_) = 4.86 × 10^2^*Q_i_* + 1.97 × 10^1^*V_h_* + 7.06 × *E_c_* + 4.09 × 10^−3^*E_nn_* − 1.65 × 10^1^ (1)

*n* = 20, *F* = 21.13, *s*^2^ = 0.0246, *R*^2^ = 0.8579, *R*^2^_cv_ = 0.7196.

The correlation coefficients (*R*^2^), cross validated correlation coefficient (*R*^2^_cv_), Fisher criterion values (*F*), regression coefficient (X) and standard errors for the regression coefficient (∆*X*) and *t*-test values corresponding to Equation (1) are given in Table 3. The *R*^2^ value of 0.8579 with the *R*^2^_cv_ value of 0.7196 showed the good predictive power of the developed model.

The charge distribution-related or electronic descriptor ($Q_{i}$, $E_{nn}$) and quantum chemical descriptor ($V_{h\;,\;}\;E_{c}$) were found to influence the activity of PTs in the QSAR equation. The *t*-test values indicated that the statistical significance of the selected descriptors in the QSAR model, decreased in the order: $V_{h}$ > $Q_{i}$ > $E_{c}$ > $E_{nn}$.

It is known that the local electron densities or charges determine the mechanism and the rate of most chemical reactions and physico-chemical properties of compounds. The valence electrons in molecules are not fixed to any particular atom but can move around the molecule. The electrons will be more at electronegative atoms compared to electropositive ones, thus resulting in the molecules being partially negative while the others partially positive [30]. In Equation (1), two electronic descriptors were involved.

**Table 3:** The best nonlinear seven descriptors selected using BML method for predicted compound (PTC) analogue training set (*R*^2^ = 0.8579, *R*^2^_cv_ = 0.7196, *F* *=* 21.13, *R*^2^ − *R*^2^_cv_ = 0.1383).

Descriptor	Symbol	*t*-Test	*X*	Δ*X*
Min partial charge for a C atom (Zefirov’s PC)	$Q_{i}$	5.8487	4.8595 × 10^2^	8.3087 × 10
Min valency of an H atom	$V_{h}$	7.1708	1.9708 × 10	2.7483 × 10^0^
Max bond order of a C atom	$E_{c}$	4.3256	7.0647 × 10^0^	1.6332 × 10^0^
Molecular surface area	$E_{nn}$	2.4697	4.0917 × 10^−3^	1.6568 × 10^−3^
Intercept		−4.4980	−1.6475 × 10	3.6628 × 10^0^

Minimum partial charge for a carbon atom (Zefirov’s PC), ($Q_{i}$) descriptor or partial charge is important for the ionic interactions between the drugs and its binding site on the receptors. The positive regression in the model in Equation (1) showed that the bigger the partial charge in the molecule, the higher the inhibition towards hyaluronidase.

Total molecular surface area ($E_{nn}$) is a combination of the contribution of atomic partial charges to the total molecular solvent-accessible surface area. This descriptor and the minimum partial charge for a carbon atom (Zefirov’s PC) ($Q_{i}$) suggest the importance of the interaction between the inhibitor molecular surface area with solvent.

Two quantum chemical descriptors were involved in the selected model. The first quantum chemical descriptor was the minimum valency of the H atom ($V_{h}$). It describes the atomic valence state for the energies of the given atomic (H) species in the molecule and its fragments [30]. It characterizes the magnitude of the perturbation experienced by an atom in the molecular environment as compared to the isolated atom.

The maximum bond order of a C atom descriptor, $E_{c}$, is categorized as a valency-related descriptor. This descriptor is related to the strength of the intramolecular bonding interactions and characterizes the stability, conformational flexibility and other valency-related properties of the molecules. This suggests the importance of the CO group for the interaction between the inhibitor and biological receptor.

From the predicted log IC_50_ values, it is clear that the QSAR equation generated through the quantum chemical method predicted that the pIC_50_ values were very close to the experimental values (Table 4).

**Table 4:** Experimental and predicted log IC_50_ values of test set compounds.

Test Set Compound	Experimental Log IC_50_	Predicted Log IC_50_	Differences	Percentage Differences
6	2.01	1.8	0.2	8.5
8	2.3	2.1	0.18	7.5
11	2.9	2.9	0.05	1.6
19	3.2	2.5	0.6	3.9

### 2.6. Design of a New Potential Pentacylic Triterpene

After repeating for seven times in designing new compounds for this pentacylic teriterpene data set, below is the structure of the new design with the highest activity, which was indicated by the smallest pIC_50_ value.

**Figure 4:**
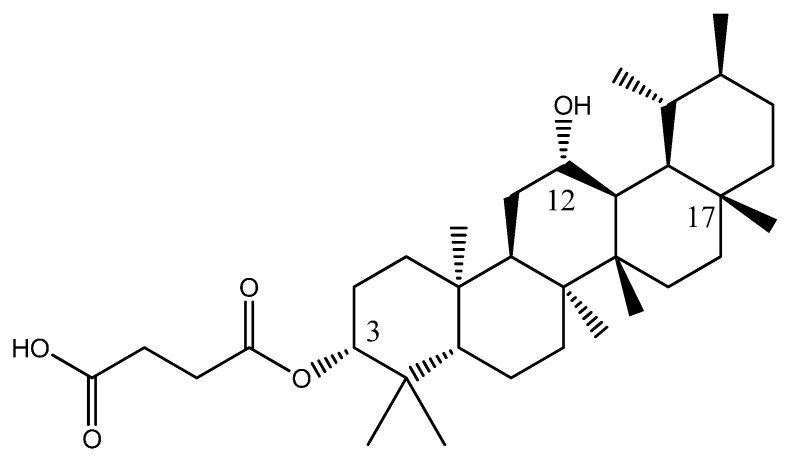
Structure of new PTC A compound.

Several structures have been designed continuously until Equation (1) gave the smallest value for log IC_50_. This new compound PTC A consist of a carboxypropionylox group, S2, a hydroxyl group at carbon-12 and a methyl group at carbon-17 which was believed to give rise to the biological activity of PTC A (Figure 4). Equation (1) provided a prediction value of pIC_50_ as 1.6183 for this compound, which was the smallest compared to compounds **1**–**23** and the other designed molecules.

### 2.7. Method Validation of the Proposed Model

The most important test of the model is its ability to correctly predict the properties of other or new compounds that were not included in the QSAR model. The leave-one-out method technique is based on the difference between the squared cross-validated correlation coefficient (*R*^2^_cv_) and the correlation coefficient (*R*^2^) [31,32]. The corresponding *R*^2^_cv_ for all selected models will be calculated automatically by the validation module, which was implemented in the CODESSA 2.6 package. The value of *R*^2^_cv_ (0.7196) was found to be close to the value of *R*^2^ (0.8579). The difference was less than 0.3, which suggested good predictive ability of the selected best multi linear model [33].

The external validation is a more reliable way to establish a predictive QSAR model [34]. The correlation coefficient prediction (*R*^2^_pred_), which was based only on the molecules present in the test set, should be more than 0.5. The value of *R*^2^_pred_ for the training set of this model was 0.5831, which was more than 0.5.

### 2.8. Possible Interactions from an in Silico Molecular Docking Study

**Figure 5:**
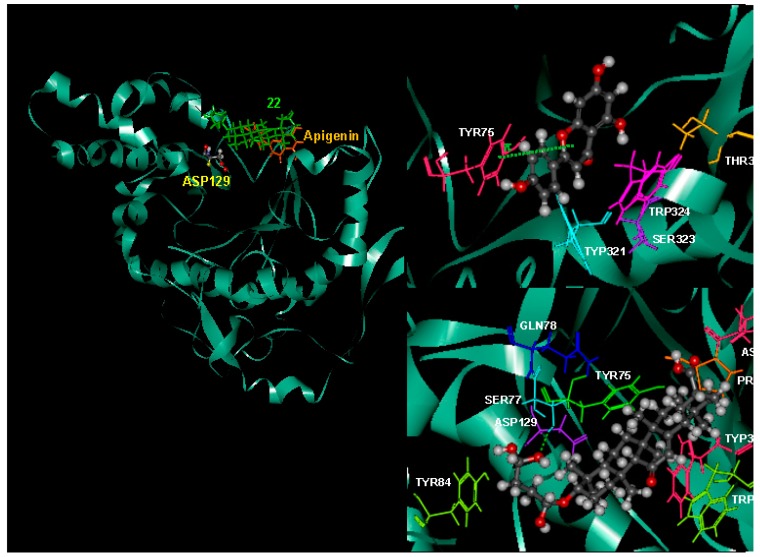
Superimposed the complex structures of apigenin and 22 with human hyaluronidase (**left**). The interactions with residues interaction energy below −4 kcal/mol of apigenin (**right**, **top**) and 22 (**right**, **bottom**) with human hyaluronidase were illustrated. The π–π and hydrogen bonding interaction are depicted in orange and green dashed, respectively.

Molecular docking study was used to clarify the binding mode and identify the interaction of inhibitors with our targeting protein, human hyaluronidase. The flexible docking result with AutoDock Vina indicated that compound **22** was more active than apigenin with the negative binding affinities range of −8.5 to −7.6, where those of apigenin was in the range of −7.8 to −7.2. The lower (negative) number indicated the stronger binding of the compound with the protein receptor. The selected docked complex was further minimized and visualized for their interactions with CHARMM force field against hyaluronidase in Discovery Studio in Figure 5. In the representation, apigenin and compound **22** in orange, and green, respectively, were superimposed to compare their interactions. A close view of the interactions has been depicted, whereas the orange and green dotted lines represented the pi–pi interactions and hydrogen bonds. The details of the van der Waals (VDW), electrostatic, binding interaction (BI) with amino acids within 4 Å vicinity of the compound and total interaction energy (IE) value are tabulated in Table 5. All the results from binding affinity with AutoDock Vina, BI with amino acid residues within 4 Å, and IE using CHARMM forcefield are in agreement with the experimental results and confirmed the stronger inhibition of compound **22** against hyaluronidase than apigenin. The residues with strong interaction energy below −4 kcal/mol for apigenin are TYR75, TRP321, SER323, TRP324, THR327, where more residue interactions with ASN61, PRO62, TYR75, SER77, GLN78, TYR84, ASP129, TRP321, TRP324 for **22** are found. Apigenin did not bind well with one of the reported binding sites. ASP129, however, the π–π interaction between apigenin with TYR75 and no hydrogen bonding were observed. Compound **22** forms two hydrogen bonding interactions with SER77 and GLY63 (SER77:HG-22:O45 and 22:H50-GLY63:O). Compound **22** bound stronger, about 50.16 kcal/mol (−191.07 to −140.91) from the total interaction energy. Detailed on the binding interaction within 4 Å from the compound is also supported at the lower BE of −88.81 and −32.09 kcal/mol for compound **22** and apigenin, respectively. The contribution of the stronger interaction of compound **22** mainly comes from the electrostatic interaction (−57.15) rather than van der Waals (−31.66 kcal/mol).

**Table 5:** Contribution of the interaction energy in kcal/mol of human hyaluronidase binding residues in the 4 Å from apigenin and **22**. Residues with strong interaction energy below −4 kcal/mol are highlighted.

Residue	Interaction Energy (IE)	VDW	Electrostatic	Residue	Interaction Energy (IE)	VDW	Electrostatic
Apigenin				22			
ALA38	−1.03	−0.64	−0.39	ASN39	−1.81	−0.77	−1.04
ASN39	−1.57	−2.05	0.48	**ASN61**	**−5.72**	**−0.56**	**−5.16**
PRO62	−2.93	−2.23	−0.71	**PRO62**	**−4.00**	**−2.89**	**−1.11**
GLY63	−1.34	−1.94	0.60	GLY63	−2.94	−1.94	−1.00
**TYR75**	**−4.22**	**−4.04**	**−0.18**	**TYR75**	**−15.15**	**−6.08**	**−9.07**
SER77	−0.69	−0.37	−0.32	SER76	−3.83	−2.84	−1.00
**TRP321**	**−4.33**	**−1.51**	**−2.82**	**SER77**	**−9.99**	**−2.00**	**−7.99**
VAL322	0.60	−0.44	1.04	**GLN78**	**−4.95**	**−0.52**	**−4.43**
**SER323**	**−4.57**	**−0.69**	**−3.88**	TYR82	−2.31	−0.72	−1.59
**TRP324**	**−7.40**	−3.80	−3.60	**TYR84**	**−5.56**	**−3.08**	**−2.48**
**THR327**	**−4.61**	**−0.53**	**−4.07**	**ASP129**	**−10.36**	**−0.86**	**−9.50**
				GLU131	−2.55	−2.34	−0.21
				**TRP321**	**−8.18**	**−1.51**	**−6.67**
				**TRP324**	**−11.45**	**−5.56**	**−5.89**
BE in 4 Å	−32.09	−18.24	−13.85	BE in 4 Å	−88.81	−31.66	−57.15
Total IE	−140.91	−23.62	−117.28	Total IE	−191.07	−39.23	−151.84

## 3. Materials and Methods

### 3.1. Chemicals and Instruments

All chemicals were obtained from commercial sources (Aldrich, Merk, and Sigma, Darmstadt, Germany) and used without further purification. Solvents were used without further purification or drying, unless stated otherwise. Reactions and isolations were monitored using thin layer chromatography (TLC) (aluminum supported silica gel 60 _F254_ plates were used for TLC). TLC spots were visualized under ultra-violet light (254 and 365 nm). The plates were then sprayed with 10% sulphuric acid followed by heating using a hot plate to detect the presence of phenolics and terpenes, which were indicated by the presence of colourful spots. Several packing materials were used for column chromatography *i.e.*, MCI gel CHP 20P, Sephadex LH-20, Chromatorex ODS, silica gel 60 (70–230 Mesh ASTM or equivalent to silica gel of size 0.063–0.200 mm). The infrared spectra were recorded on a Perkin Elmer Spectrum 100 Fourier Transform Infrared (FT-IR) spectrometer (Perkin Elmer, Waltham, MA, USA) equipped with a mid-infrared deuterated triglycine sulphate (DTGS) detector. NMR analyses were carried out on a Bruker DRX 300 NMR spectrometer (300 MHz for ^1^H NMR and 75 MHz for ^13^C NMR, Bruker corporation, Billerica, MA, USA) system with deuterated pyridine (C_5_D_5_N). The mass spectra were obtained using an LTQ Orbitrap Mass spectrometer (Thermo Fisher Scientific, Bremen, Germany) equipped with an electrospray ionization probe by employing either a negative or positive ion mode, whichever could afford the best limits of detection for the compounds.

### 3.2. Plant Material

*P. tetrandra* was collected from Setiu, Terengganu on the 29 January 2006 and was deposited in the herbarium of the Forest Research Institute Malaysia (FRIM) with a herbarium specimen number of FRI 50080. The sample was identified by an FRIM botanist. The plant material was dried in an oven at 40 °C, divided into different parts and ground.

### 3.3. Extraction and Isolation

Oven-dried leaves (2.041 kg) were ground and extracted with 5 L of methanol (MeOH) by soaking three times at room temperature. The concentrated extract (20.1 g) was suspended in H_2_O (14 L) and partitioned with petroleum ether (5 L, 3×) followed by chloroform (CHCl_3_) (5 L, 3×) and finally with ethyl acetate (EtOAc) (5 L, 3×). The CHCl_3_ layer (15.1 g) obtained was concentrated and chromatographed over a Diaion HP-20SS column using 100% MeOH to afford three fractions. Fractions 1 and 2 were combined and purified using a silica gel column eluting with a CHCl_3_-MeOH (CM) eluent system, to afford 3β,19,23-trihydroxyurs-12-en-28-oic acid **2** (20 mg, 96:4 ***v***/***v***). Fraction 3 was also further fractionated and purified using silica gel column chromatography eluting with a CHCl_3_-MeOH (CM) eluent system to give 3β-hydroxyurs-12-en-28-oic acid (ursolic acid) **1** (1.5 g 98:2 ***v***/***v***).

Sub-fraction C3 (2.79 g), which highly inhibited the activity of the hyaluronidase enzyme was further fractionated and purified using silica gel column chromatography (chloroform: methanol; 10:0 → 9:1 *v*/*v*) to give eight sub-fractions (C3-1 to C3-8). Sub-fraction 6 (C3-6) exhibited the highest inhibition against the hyaluronidase enzyme and TPA induced mouse ear oedema activity. Column chromatography of this fraction with chloroform: methanol (98:2 *v*/*v*) yielded 3β-hydroxyurs-12-en-28-oic acid (ursolic acid) **1** (1.5 g, 98:2).

The cut and oven-dried roots (2.2 kg) were ground and extracted with 5 L of MeOH by soaking three times at room temperature. The concentrated extract (100 g) was suspended in H_2_O and partitioned with EtOAc (5 L, 3×). The EtOAc layer after drying under reduced pressure (15.6 g) was chromatographed over silica gel and eluted using petroleum ether with increasing amounts of acetone to give eight fractions. Fraction 3 was further fractionated and purified by a silica gel column eluting with a hexane-CHCl_3_ eluent system to afford 3β-acetylolean-12-en-28-oic acid **3** (20 mg, 100:0 ***v***/***v***).

3β*-*Urs*-*12*-*en*-*28*-*oic acid **1** 1.5 g, ^1^H NMR (300 MHz, C_5_D_5_N): δ (ppm) 0.95 (1H, *m*, H-1α), 1.30 (1H, *m*, H-1β), 1.80 (2H, *m*, H-2), 3.50 (1H, *brt*, *J* = 8.5 Hz, H-3), 1.10 (1H, *m*, H-5), 1.50 (2H, *m*, H-6), 1.60 (2H, *m*, H-7), 1.55 (1H, *m*, H-9), 1.90 (2H, *m*, H-11), 5.54 (1H, *s*, H-12), 1.15, 2.30 (2H, *m*, H-15), 2.19 (2H, *dt*, *J* = 4.4, 8.6 Hz, H-16), 2.68 (1H, *d*, *J* = 11.8 Hz, H-18), 1.25 (1H, *m*, H-19), 1.00 (1H, *m*, H-20), 1.50 (2H, *m*, H-21), 1.75 (2H, *m*, H-22), 1.25 (3H, *s*, H-23), 1.04 (3H, *s*, H-24), 0.90 (3H, *s*, H-25), 1.08 (3H, *s*, H-26), 1.28 (3H, *s*, H-27), 1.03 (3H, *d*, *J* = 4.8 Hz, H-29), 0.99 (3H, *d*, *J* = 5.2 Hz, H-30). ^13^C NMR (75 MHz, C_5_D_5_N): δ (ppm) 38.9, 28.4, 77.7, 39.3, 55.6, 18.5, 33.3, 39.8, 47.9, 37.2, 23.4, 125.5, 139.1, 42.3, 27.8, 24.7, 47.9, 53.3, 39.2, 39.2, 30.8, 37.0, 28.6, 16.4, 15.5, 17.2, 23.7, 180.0, 17.3, 21.2.

3α,19α,24*-*Trihydroxyurs*-*12*-*en*-*28*-*oic acid **2** 20.0 mg, ^1^H NMR (300 MHz, C_5_D_5_N): δ (ppm): 1.35 (1H, *m*, H-1), 1.8 (2H, *m*, H2), 4.50 (1H, *bs*, H-3), 1.80 (1H, *m*, H-5), 1.75 (1H, *m*, H-6), 1.2 (1H, *m*, H-7), 1.4 (1H, *m*, H-9), 2.2 (1H, *m*, H-11), 5.64 (1H, *s*, H-12), 2.14 (1H, *m*, H-15), 3.14 (2H, *dt*, *J* = 12.6, 4.0 Hz, H-16), 3.02 (1H, *s*, H-18), 2.3 (1H, *m*, H-20), 2.1 (2H, *m*, H-21), 2.5 (2H, *m*, H-22), 1.65 (3H, *s*, H-23), 4.13 (1H, *d*, *J* = 10.8 Hz, H-24), 3.84 (1H, *d*, *J* = 10.8 Hz, H-24), 1.0 (3H, *s*, H-25), 1.14 (3H, *s*, H-26), 1.68 (3H, *s*, H-27), 1.46 (3H, *s*, H-29), 1.16 (3H, *d*, *J* = 9.6 Hz, H-30). ^13^C NMR (75 MHz, C_5_D_5_N): δ (ppm) 33.8, 26.7, 69.7, 43.7, 48.1, 18.9, 33.6, 41.8, 47.5, 37.2, 24.0, 128.0, 139.7, 40.3, 29.05, 26.2, 47.5, 54.4, 72.5, 42.2, 26.2, 38.3, 23.4, 65.5, 15.8, 16.9, 24.4, 180.6, 26.9, 16.6.

3β*-*Acetylolean*-*12*-*en*-*28*-*oic acid **3** 20 mg, ^1^H NMR (300 MHz, C_5_D_5_N): δ (ppm): 1.50, 1.15 (2H, *m*, H-1), 1.45 (2H, *m*, H-2), 4.73 (1H, *dd*, *J* = 5.0, 11.1 Hz), 1.00 (1H, *m*, H-5), 1.50 (1H, *m*, H-6), 1.65 (1H, *m*, H-7), 1.20 (1H, *m*, H-9), 1.85 (2H, *m*, H-11) , 5.5 (1H, *bs*, H-12), 1.40 (2H, *m*, H-15), 2.10 (1H, *m*, H-16β), 1.90 (1H, *m*, H-16α), 3.35 (1H, *dd*, *J* = 4.6, 13.3 Hz, H-18), 1.91 (2H, *m*, H-19), 1.50 (2H, *m*, H-21), 1.90 (2H, *m*, H-22), 0.94 (3H, *s*, H-23), 0.86 (3H, *s*, H-24), 0.91 (3H, *s*, H-25), 0.97 (3H, *s*, H-26), 1.30 (3H, *s*, H-27), 1.02 (3H, *s*, H-29), 1.04 (3H, *s*, H-30), 2.07 (3H, *s*, COCH_3_). ^13^C NMR (75 MHz, C_5_D_5_N): δ (ppm) 38.0, 23.5, 80.5, 37.6, 55.3, 18.2, 33.0, 39.4, 47.6, 36.9, 23.4, 123.6, 139.0, 41.7, 28.0, 23.6, 46.2, 41.0, 46.4, 30.7, 33.9, 32.8, 27.9, 16.7, 15.1, 17.1, 25.9, 180.1, 33.0, 23.5, 20.9, 170.5.

### 3.4. Synthesis of Ursolic Acid Analogues

Ursolic acid **1** was selected as the lead compound and seven derivatives **4**–**10** were prepared from the semisynthesis of it. The modification methods were carried out as described by Ma *et al.* [16], with a slight modification in the solvents and reagents used. The reaction scheme used for the synthesis of the ursolic acid **1** derivatives is shown in Figure 1.

#### 3.4.1. 3-Oxo-urs-12-en-28-oic Acid (**4**)

Pyridinium chlochromate (PCC) (161.3 mg, 0.75 mmol) was added to a solution of ursolic acid **1** (112.3 mg, 0.25 mmol) in a acetone-dicholoromethane (5:5, 10 mL) mixture. After stirring at room temperature until the reaction was almost complete (monitored with TLC), the mixture was concentrated and partitioned with H_2_O and CH_2_Cl_2_. The CH_2_Cl_2_ layer was concentrated and purified by silica gel column chromatography eluting with hexane: acetone (95:5 *v*/*v*) to give **4** Yield 79.34%, white amorphous powder.

^1^H NMR (300 MHz, C_5_D_5_N): δ (ppm) 5.60 (1H, *bs*, H-12), 2.15 (2H, *dt*, *J* *=* 4.4, 8.6 Hz, H-16), 2.75 (1H, *d*, *J* = 11.8 Hz, H-18), 1.04 (3H, *s*, H-23), 0.99 (3H, *s*, H-24), 0.90 (3H, *s*, H-25), 1.02 (3H, *s*, H-26), 1.16 (3H, *s*, H-27), 1.22 (3H, *s*, H-29), 1.00 (3H, *s*, H-30). ^13^C NMR (75 MHz, C_5_D_5_N): δ (ppm) 38.7, 28.0, 218.0, 47.5, 54.7, 19.2, 33.7, 39.2, 46.5, 36.8, 23.3, 126.0, 140.0, 41.9, 30.4, 24.2, 46.1, 53.0, 38.9, 38.8, 32.3, 36.2, 26.1, 16.9, 14.5, 16.7, 23.3, 182.0, 20.8, 21.0.

#### 3.4.2. 3-Hydroxyimino-urs-12-en-28-oic Acid (**5**)

A solution of **4** (26.2 mg, 0.34 mmol) and hydroxylamine hydrocholoride (27.6 mg, 0.40 mmol) in pyridine (5 mL) was heated for four hours at 50 °C. After cooling to room temperature, the reaction mixture was concentrated under vacuum to dryness. It was then purified over a silica gel column eluted with petroleum ether: CHCl_3_ (95:5 *v*/*v*) to obtain **5** Yield 90%, colorless crystal.

^1^H NMR (300 MHz, C_5_D_5_N): δ (ppm) 3.5 (2H, *bd*, *J* = 15.5 Hz), 2.6 (*bd*, *J* = 11.25 Hz, H-2), 5.50 (1H, *bs*, H-12), 2.30 (1H, *m*, H-18), 1.08 (3H, *s*, H-23), 0.92 (3H, *s*, H-24), 0.91 (3H, *s*, H-25), 0.98 (3H, *s*, H-26), 1.20, (3H, *s*, H-27), 1.40 (3H, *s*, H-29), 0.96 (3H, *s*, H-30). ^13^C NMR (75 MHz, C_5_D_5_N): δ (ppm) 38.9, 28.8, 164.4, 40.1, 56.4, 19.5, 33.4, 40.3, 47.6, 37.6, 23.8, 125.7, 139.4, 42.7, 25.0, 48.2, 53.7, 37.4, 39.6, 31.2, 37.4, 28.2, 21.6, 15.2, 17.5, 23.9, 180.2, 21.6, 17.7.

#### 3.4.3. 3-Acetyl-urs-12-en-28-oic Acid (**6**)

Ursolic acid **1** (120.0 mg, 0.26 mmol) was treated overnight with acetic anhydride (534.0 mg, 5.32 mmol) and pyridine at room temperature and worked up with 10% HCl, NaHCO_3_, followed by separation using a separating funnel to get the CH_2_Cl_2_ layer. MgSO_4_ was added to absorb water from it. The solution was filtered and rinsed using CH_2_Cl_2_. It was purified over a silica gel column eluted using chloroform: petroleum ether (90:10 *v*/*v*) to give **6**. Yield 50%, white amorphous powder.

^1^H NMR (300 MHz, C_5_D_5_N): δ (ppm) 4.6 (1H, *dd*, *J* = 5.06, 10.9 Hz, H-3), 5.45 (1H, *bs*, H-12), 2.24 (2H, *m*, H-16), 2.65 (1H, *d*, *J* = 11.1 Hz, H-18), 1.06 (3H, *s*, H-23), 0.91 (3H, *s*, H-24), 0.88 (3H, *s*, H-25), 0.99 (3H, *s*, H-26), 1.08 (3H, *s*, H-27), 1.23 (3H, *s*, H-29), 0.95 (3H, *s*, H-30), 2.09 (3H, *s*, COCH_3_). ^13^C NMR (75 MHz, C_5_D_5_N): δ (ppm) 38.8, 28.0, 79.1, 41.8, 54.9, 17.9, 36.5, 38.9, 47.4, 36.8, 22.9, 124.8, 138.6, 41.9, 32.7, 24.3, 47.2, 52.9, 37.8, 39.3, 30.5, 37.3, 27.6, 16.4, 15.0, 16.9, 23.3, 179.3, 16.8, 20.6, 20.9, 169.9.

#### 3.4.4. 3-Hydroxy-urs-12-en-28-oic Acid Methyl Ester (**9**)

To a stirred solution of ursolic acid **1** (10.0 mg, 0.02 mmol) in approximately 10 mL of toluene: MeOH (3:2), a solution of TMSCHN_2_ (trimethylsilane diazomethane) in hexane was added drop wise until the yellow color persisted. The mixture was stirred at room temperature and concentrated. It was purified over a silica gel column with hexane: CHCl_3_ (70:30 *v*/*v*) as the eluent to give **9**. Yield 95%, white amorphous powder.

^1^H NMR (300 MHz, C_5_D_5_N): δ (ppm) 3.51 (1H, *brt*, H-3), 2.62 (1H, *d*, *J* = 13.11 Hz, H-18), 1.10 (3H, *s*, H-23), 0.99 (3H, *s*, H-24), 0.92 (3H, *s*, H-25), 0.98 (3H, *s*, H-26), 1.00 (3H, *s*, H-27), 1.30 (3H, *s*, H-29), 1.20 (3H, *s*, H-30), 3.75 (3H, *s*, COOCH_3_). ^13^C NMR (75 MHz, C_5_D_5_N): δ (ppm) 39.8, 28.8, 78.0, 39.0, 55.7, 18.7, 33.3, 39.6, 47.9, 37.3, 24.5, 125.9, 138.7, 42.2, 28.7, 23.3, 51.4, 53.3, 39.3, 39.2, 30.7, 36.9, 28.1, 16.5, 15.6, 17.2, 22.9, 177.7, 21.2, 17.3.

#### 3.4.5. 3-Oxo-urs-12-en-28-oic Acid Methyl Ester (**7**)

Methyl ursolate **9** was treated with PCC in the same manner as was carried out for compound **4** to obtain compound **7**. The resultant was purified over a silica gel column eluted with chloroform: petroleum ether (90:10 *v*/*v*). Yield 90%, colorless crystal.

^1^H NMR (300 MHz, C_5_D_5_N) δ (ppm): 5.42 (1H, *s*, H-12), 2.49 (1H, *d*, *J* = 10.8 Hz, H-18), 0.98 (3H, *s*, H-23), 0.97 (3H, *s*, H-24), 0.90 (3H, *s*, H-25), 1.13 (3H, *s*, H-26), 1.24 (3H, *s*, H-27), 1.26 (3H, *s*, H-29), 0.96 (3H, *s*, H-30), 3.75 (3H, *s*, COOCH_3_). ^13^C NMR (75 MHz, C_5_D_5_N): δ (ppm) 39.9, 28.5, 216.3, 48.4, 55.4, 21.3, 34.5, 47.1, 42.5, 37.1, 24.6, 125.8, 138.9, 42.5, 26.8, 23.7, 47.5, 53.5, 39.4, 39.3, 32.8, 36.9, 30.9, 17.2, 15.3, 17.4, 48.7, 177.8, 19.9, 21.7, 23.9.

#### 3.4.6. 3-Hydroxyimino-urs-12-en-28-oic Acid Methyl Ester (**8**)

A solution of **7** (80.0 mg, 0.17 mmol) and hydroxylamine hydrochloride (108.0 mg, 1.56 mmol) in pyridine was heated for 2 h at 50 °C. It was then cooled to room temperature and diluted with CH_2_Cl_2_ followed by washing with 10% HCl (3×). It was then dried over anhydrous Na_2_SO_4_ and concentrated under reduced pressure. It was purified over a silica gel column using CHCl_3_: hexane (40:60 *v*/*v*) as the eluent to give **8** Yield 95%, white amorphous powder.

^1^H NMR (300 MHz, C_5_D_5_N): δ (ppm) 3.50 (1H, *d*, *J* = 15.58 Hz, H-2β), 2.25 (1H, *m*, H-2α), 5.35 (1H, *s*, H-12), 2.30 (2H, *m*, H-16), 2.42 (1H, *d*, *J* = 10.8 Hz, H-18), 0.97 (3H, *s*, H-23), 0.90 (3H, *s*, H-24), 0.85 (3H, *s*, H-25), 1.12, (3H, *s*, H-26), 1.10 (3H, *s*, H-27), 1.41 (3H, *s*, H-29), 0.92 (3H, *s*, H-30), 3.68 (3H, *s*, COOCH_3_), 12.36 (1H, *s*, NOH). ^13^C NMR (75 MHz, C_5_D_5_N): δ (ppm) 39.3, 24.7, 164.2, 38.9, 56.4, 19.4, 33.2, 39.4, 47.5, 37.1, 23.7, 126.0, 138.9, 42.5, 28.5, 24.7, 48.4, 53.5, 40.0, 40.3, 30.9, 37.3, 28.1, 17.4, 15.2, 17.5, 23.9, 177, 51.6, 21.4, 17.0.

#### 3.4.7. 3-Acetyl-urs-12-en-28-oic Acid Methyl Ester (**10**)

Compound **9** was treated with TMSCHN_2_ using the similar method, which was used for the preparation of **9** to give **10**. Yield 95%, colorless crystal.

^1^H NMR (300 MHz, C_5_D_5_N): δ (ppm) 4.75 (1H, *dd*, *J* = 5.4, 10.8 Hz, H-3), 5.41 (1H, *s*, H-12), 2.49 (1H, *d*, *J* = 11.1 Hz, H-16), 1.02 (3H, *s*, H-23), 0.98 (3H, *s*, H-24), 0.94 (3H, *s*, H-25), 1.00 (3H, *s*, H-26), 1.04 (3H, *s*, H-27), 1.20 (3H, *s*, H-29), 0.96 (3H, *s*, H-30), 2.09 (3H, *s*, COCH_3_), 3.74 (3H, *s*, COOCH_3_). ^13^C NMR (75 MHz, C_5_D_5_N): δ (ppm) 38.4, 28.5, 80.8, 39.3, 55.6, 18.5, 33.3, 38.0, 47.8, 38.0, 23.0, 125.8, 138.8, 39.3, 28.5, 24.7, 51.6, 53.4, 42.4, 39.9, 30.9, 37.1, 28.3, 17.1, 15.6, 17.2, 24.0, 177.8, 17.4, 21.2, 170.7, 21.4, 24.0.

### 3.5. Hyaluronidase Inhibitory Assay

The assay was performed following the modified Sigma protocol [27].

### 3.6. Construction of a QSAR Model

All the molecular structures were built by the Chem3D Ultra package, and the structures were optimized using the MM2 force field. The lowest energy conformations obtained by molecular mechanics calculations were optimized by the quantum chemical semi empirical RM1 (Recife Model 1) method [35]. The RM1 method was selected for our calculations because the average errors in the prediction of enthalpies of formation, dipole moments, ionization potentials, and inter atomic distances, using the RM1 methods were found to be less than the average errors given by AM1, PM3 and PM5 methods. The MOPAC program [36] was used to do semi empirical molecular orbital calculations, by passing the RM1 parameters via the keyword EXTERNAL in MOPAC along the keyword AM1. The optimized structures were found to be in good agreement with the available crystal structure of **10** reported earlier [37].

An input file, which contained the data obtained from self-consistent field (SCF), thermodynamics, force and molecular structure calculations for each structure together with the activity value (log IC_50_) was prepared. All the data files were loaded into the CODESSA 2.6 for further calculation of topological, conventional, geometrical, electrostatic, quantum chemical and thermodynamic descriptors. More than 450 descriptors could be calculated from this program. The descriptors were further analyzed for linear dependence.

The good statistical methods that could select appropriate descriptors and the best quality correlation are essential in developing the QSAR/QSPR models. In this study, two methods were used to obtain the QSAR equation *i.e.*, Heuristic and the Best Multi Linear regression method (BML). The Heuristic model could work fast and could be applied on a no limit data set. It could either give good correlation from the data or several best regression models. The algorithm of this method follows several steps, summarizing as it eliminates the descriptors with bad or missing values followed by the highly intercorrelated descriptors. The best multi-parameter regression models, which were developed from the remaining descriptors, will come with optimum values of statistical criteria consisting of regression correlation coefficient (*R*^2^), the cross-validation (*R*^2^_cv_), and the *F*-value. Compared to the Heuristic method, the BML method was more thorough and thus takes a longer time to complete. This method also limits the number of experimental values (150 structures) and descriptors (300 descriptors). The best two, three and *etc.* parameter regression models were based on the highest *R^2^* value. The models will be constituted from the selected non-collinear descriptors. After the initial analysis, the equation from BML was selected as the best equation based on the statistical parameters such as correlation coefficient (*R*), standard deviation (*s*), and *F* values. The BML method builds a single correlation using all selected descriptors in order to find the best regression model.

In developing a good QSAR model, it is important to decide when to stop adding descriptors. The technique is the so-called “breaking point” which helps to control the model expansion and thus, in turn, improves the statistical quality of the model [28]. Initially, any addition of any independent variables in the QSAR model can lead to improvement in the *R*^2^ value in the consequent regression. However, if the addition of the new descriptor does not significantly improve the *R*^2^ value, then the added descriptor does not contribute any new information to the model. If the increase in *R*^2^ value is less than 0.02, then the QSAR model described by such regression equation is considered as the best model.

The obtained model was validated to test the internal stability and predictive ability by using the internal test procedure. It employed the leave-one-out method (LOD). In the calculation for the cross validation regression coefficient (*R*^2^_cv_), each molecule in the data set was eliminated once. The activity of the eliminated molecule was predicted by using the model developed by the remaining molecules. The cross validation regression coefficient (*R*^2^_cv_) can be calculated by the following formula [38];

(2) $$R_{\text{cv}}^{2}=1-\frac{\sum {\left(Y_{\text{pred}}-Y_{\exp}\right)}^{2}}{\sum {\left(Y_{\exp}-\bar{Y}\right)}^{2}}$$

where *Y*_exp_ and *Y*_pred_ were activities of molecule in the test data and $\bar{Y}$ is the average activity of all the molecules in the data set. The LOD method technique is based on the difference between the squared cross-validated correlation coefficient (*R*^2^_cv_) and correlation coefficient (*R*^2^). The small difference suggested a good predictive ability of the QSAR model.

When the data set is divided into the training and test sets, a model is generated from the training set compounds. The model should be validated through the external validation using the parameters like *R*^2^_pred_. It could be defined as

(3) $$R_{\text{pred}\;}^{2}=1-\;\frac{\sum^{\text{​}}{\left(Y_{\text{pred }\left(\text{test}\right)}-Y_{\text{test}}\right)}^{2}}{\sum^{\text{​}}{\left(Y_{\text{test}}-Y_{\text{training}}\right)}^{2}}$$

The value of *R*^2^_pred_ should be more than 0.5.

#### 3.6.1. Data Set

A total of 24 anti-inflammatory compounds were used to develop the QSAR model. Out of these compounds, compounds **1**, **2** and **3** were isolated from *P. tetrandra*, compounds **4**–**10** were from the semi synthesis of ursolic acid **1**, while compounds **11**–**30** were purchased from Chromadex. All compounds were evaluated for their inflammatory activity of the hyaluronidase enzyme, and the data was calculated as IC_50_ values. The data was converted into log IC_50_ values which were used instead of IC_50_ to improve the normal distribution of the experimental data points [28].

#### 3.6.2. Descriptors

Four descriptors were found to influence the activity of PTs towards the hyaluronidase inhibitory activity. Two electronic descriptors were involved in Equation (1). The first electronic descriptor was the min partial charge for a carbon atom (Zefirov’s PC) ($Q_{i}$). It could be defined as [39]:

(4) $$Q_{i}=f\left(X_{i}\right)$$

whereby $X_{i}$ is the atomic electronegativity given by;

(5) $$X_{i}={\left(X_{i}^{0}\prod_{k=1}^{n}X_{k}\right)}^{1/(n+1)}$$

whereby $X_{i}^{0}$ is the electronegativity of the isolated atoms and *n* is the number of atoms in the first coordination sphere of a given atom “*i*”.

The other electronic descriptor was the molecular surface area, $E_{nn}$.

Two chemical descriptors were involved in the selected model. Max bond order of a C atom, $E_{c}$ is defined as [30]:

(6) $$E_{c}=\overset{occ}{\underset{i=1}{\sum }}\underset{\mu \epsilon A}{\sum }\underset{v\in B}{\sum }nic_{\text{iu}}c_{\text{jv}}$$

whereby the first summation is performed over all occupied molecular orbitals (*ni* denotes the occupation number of the *i*th MO), and the two other summations over µ and υ, the atomic orbitals belonging to the C atoms in the molecule. MO coefficients are denoted as *c*_iu_ and *c*_jv_.

The min valency of an H atom, the final quantum chemical descriptor is the max electron-electron repulsion for a C-H bond ($E_{ee}$). It could be defined as [30]:

(7) $$E_{ee}(AB)=\sum_{\mu ,v\in A}\sum_{\lambda ,\sigma \in B}P_{\mu v}P_{\lambda \sigma }\langle \mu v|\lambda \sigma \rangle$$

where $P_{\lambda \sigma }$ and $P_{\mu v}$ are the densities of the matrix element over atomic basis {μvλσ} and 〈μv|λσ〉 is the electron repulsion integrals on atomic basis {μvλσ}.

### 3.7. Molecular Docking Study

Molecular docking was used to predict and clarify the interaction of the complex between the most active ursolic acid analogue, compound **22** in Table 1, and hyaluronidase in comparison to the positive control apigenin. Apigenin was taken from the RCSB protein data bank (PDB ID = 4HKK, [40,41] where compound **22** was further modeled from apigenin and optimized with density functional theory under b3lyp/6-311g(d,p) basis set using Gaussian09 [42]. The high resolution of the crystal structure of human hyaluronidase-1, a hyaluronan hydrolyzing enzyme involved in tumor growth and angiogenesis was obtained from the RCSB protein data bank (PDB ID = 2PE4) [41,43]. The waters and ligands were removed from the original crystal structure. Then, the initial structure was modified according to the CHARMM force field with partial charge Momany–Rone [44] and minimization of the structures was performed with RMS gradient tolerance of 0.1000 kcal/(mol × Angstrom) satisfied. Flexible docking of compound **22** and apigenin into the targets was performed using AUTODOCK VINA [45] to Asp 129 (one of the binding sites) of human hyalurodinase. A 30 × 30 × 30 Å point grid was used. The low free energy complex structures were further minimized and analyzed. Detailed interaction energy was investigated by calculating binding energies using the protocol from Discovery Studio (Accelry Inc., San Diego, CA, USA, 2.5.5). This enabled us to estimate the residue interaction energy between the hyaluronidase and compounds.

## 4. Conclusions

A series of structurally related PTs were developed from the semi synthesis of ursolic acid **1**. Together with the isolated and commercial sources of the derivatives, a total of 30 compounds/derivatives were evaluated for their inhibition towards hyaluronidase. However, only the data from 20 compounds were used to develop the QSAR model using the quantum chemical approach together with statistical analysis.

Equation (1) provided a measure of the influence of the changes in the size, shape, intermolecular hydrogen bonding, and the binding of the H, C–O and C–H atoms of the pentacylic triterpenes investigated here on the inhibition towards hyaluronidase. This QSAR equation can be used to predict the hyaluronidase inhibitory activity of new PT compounds, thus providing an efficient approach to design and development of new bioactive PT compounds.

## Abbreviations

QSAR: Quantitative structure-activity relationship

BML: best multi linear model

HA: hyaluronic acid (HA)

PT: pentacylic triterpenoids

NMR: Nuclear magnetic resonance

ESI-MS: Electrospray ionization-mass spectrometry

SAR: Structure activity relationship

PTC: pentacyclic triterpene

VDW: van der Waals

BI: binding interaction

IE: interaction energy

TLC: thin layer chromatography

FT-IR: Fourier Transform Infrared

DTGS: deuterated triglycine sulphate

MeOH: methanol

CHCl_3_: chloroform

EtOAc: ethyl acetate

RM1: Recife Model 1
